# Gender board diversity across Europe throughout four decades

**DOI:** 10.1038/s41597-024-03181-8

**Published:** 2024-05-31

**Authors:** Hubert Drazkowski, Joanna Tyrowicz, Sebastian Zalas

**Affiliations:** 1 FAME|GRAPE, Warsaw, 00-660 Poland; 2 University of Warsaw, Warsaw, 00-927 Poland; 3 University of Regensburg, Regensburg, 93053 Germany; 4 IZA, 53113 Bonn, Germany

**Keywords:** Economics, Society, Interdisciplinary studies

## Abstract

We present a Gender Board Diversity Dataset (GBDD), which provides a cross-country perspective on women in management and supervisory boards that spans between 1985 and 2020. The data covers 43 European countries and accounts for private companies in addition to the stock-listed ones. GBBD was created using firm-level Orbis data. Our measures are based on a sample of more than 28 million unique firms observed for nearly seven years on average and reporting data about nearly 59 million individuals on management and supervisory boards. We provide the measures at the level of industry, country and year (firm-level data is proprietary). We provide three measures. The first is the share of women among all board members in a given industry, country, and year. The second one is the average of the shares of women across firms in a given industry, country and year. We also provide a new measure: the share of firms in a given industry, country and year which report no single woman on their board(s).

## Background & Summary

This document describes Gender Board Diversity Dataset (GBDD). Typically, in cross-country contexts, gender board diversity is studied with data limited to public (listed) companies, whereas the majority of board positions, as well as the majority of jobs in general, are in private (not listed) companies. Data concerning private companies are scarce. Admittedly, in a few countries, registry data were made available for research (e.g. Norway, France, Italy). GBDD is the first data to provide a comprehensive and comparable overview of gender board diversity in 43 countries spanning 36 years.

We obtain GBDD by harmonizing the firm-level data circulated as Orbis. This data source is uniquely suitable in two key ways. First, it provides an overview of registry and financial data for private companies, in addition to the public ones. Second, it reports the names and surnames of individuals in the executive and non-executive positions, thus allowing to move the analysis from firm-level to individual-level data. These exclusive advantages come with numerous challenges. Orbis data is notoriously difficult to process: it comes from local registries and there are important differences across countries, time and legal forms concerning what information is to be reported and how it is reported. Previously^1,2^, provide guidelines on constructing longitudinal firm-level data a for European countries using financial statements from the Orbis database^1^. work with one wave of Orbis data, whereas^2^ provide guidelines on how to work with the so-called Orbis Historical Data (OHD) released recently by Orbis.

Furthermore, gender identification is scarce in the data. For example^3^, use roughly 4% of the sample. We provide a novel method which allows us to circumvent this limitation: we use the names of the individuals and construct heuristics from linguistic rules. In addition, we demonstrate that Orbis is not fully reliable for board assignment. We provide an improvement drawing on verbal descriptions of individual positions. To the best of our knowledge, none of these have been previously pursued in the literature.

We provide three measures of gender board diversity. The first is the share of women among all board members in a given industry, country, and year. The second one is the average of the shares of women across firms in a given industry, country and year. These two measures were previously used in the literature. They can be dubbed the measures of presence. We also provide a new measure: the share of firms in a given industry, country and year which report no single woman on their board(s). This third measure relates to the absence of women, rather than their presence. The three measures are obtained for the management (executive) board, supervisory (non-executive boards) and the measure which combines management, supervisory and ambiguous boards jointly.

Our measures are based on a sample of more than 28 million unique firms observed for nearly seven years on average and reporting data about nearly 59 million individuals on management and supervisory boards. The firm-level and the person-level data is proprietary, and we cannot share it, however we provide the measures at the level of industry (two-digit NACE codes), country and year Table 1.

**Table 1:** GBDD: descriptive statistics.

Statistic	Management Board	Supervisory Board	All board positions
	Average Share		
N	60,301.00	30,478.00	64,227.00
Mean	0.20	0.21	0.21
Std. Dev.	0.17	0.21	0.14
25th perc.	0.09	0.05	0.13
Median	0.17	0.18	0.19
75th perc.	0.27	0.30	0.28
	Weighted Share		
N	60,301.00	30,478.00	64,227.00
Mean	0.20	0.21	0.22
Std. Dev.	0.16	0.20	0.13
25th perc	0.10	0.06	0.13
Median	0.18	0.19	0.20
75th perc	0.27	0.30	0.28
	Firms with no women		
N	60,301.00	30,478.00	64,227.00
Mean	0.74	0.62	0.67
Std. Dev.	0.21	0.31	0.20
25th perc.	0.65	0.45	0.55
Median	0.78	0.67	0.69
75th perc.	0.88	0.90	0.80

**Fig. 1:**
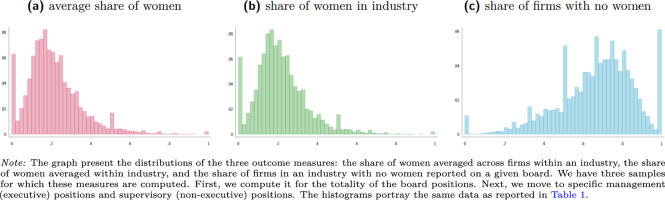
Distribution of gender board diversity measures - all board positions.

**Fig. 2:**
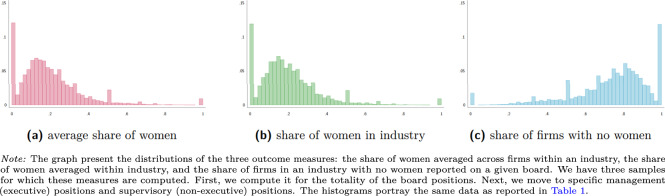
Distribution of gender board diversity measures - management board.

**Fig. 3:**
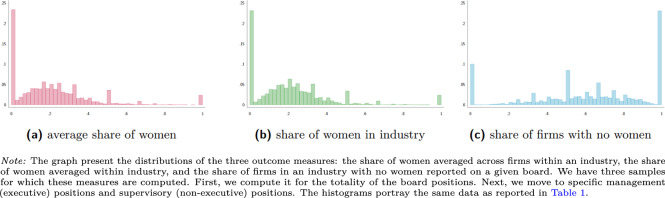
Distribution of gender board diversity measures - supervisory board.

Figures 1–3 along with Table 1 report on the properties of our data. Our data covers roughly 60 thousand observations for most measures and roughly 30 thousand observations for measures concerning specifically the supervisory boards. The mean share of women averaged across firms, and averaged within an industry range between 10% and nearly 30%. The share of industries, where no firm has a single woman in the board, average 74% for the management board and 63% for the supervisory board positions. We also calculate measures of gender diversity at the country level for stock-listed and private (non-listed) companies. The mean share of women ranges across time and countries from 10% and 30% for private companies and between 5% and slightly over 25% for stock-listed companies.

Given the massive size of our samples, our distributions are relatively smooth. Nonetheless, we see several spikes: at 0%, at 50% and at 100%. The distribution of the share of firms with no women is a less smooth, because it depends on the number of firms in a given four-digit industry rather than a number of board members and a number of women among them. The spikes at 0% for the measures of presence and at 100% for the measures of absence the fact that in relatively many firms and sectors, there are no women among the board members.

## Methods

We concatenate 11 waves of the Orbis as well as the OHD. In the past, each of the Orbis waves provided records for up to ten years for each firm. The OHD was intended to be a comprehensive compilation of the past waves. Indeed, for some firms, historical data go back as far as the early 1980s. Unfortunately, the historical data provide much fewer data points than the previously released waves. In fact, the overlap between the historical data and the data historically provided by Orbis is surprisingly low. On the upside, the historical data provides updates for firms from the 2016 wave. Combining subsequent waves ushers in many new challenges that were absent in previous studies, such as the harmonization of industry codes.

Ultimately, in addition the Gender Board Diversity Dataset (GBDD), we also present the codes that harmonize Orbis data and identify board members as well as their gender. The GBDD provides cross-country perspective on women in management and supervisory boards that spans 36 years (between 1985 and 2020). The data covers 43 European countries and accounts for private companies in addition to the listed (public) ones.

This section is structured as follows. After presenting the GBDD, we move to the details on how we obtained these measures. We start by describing the preprocessing of the data in section section Preprocessing Orbis data. In section Legal form, we provide explanations on how we address the issue of legal forms in our sample and their role in determining corporate governance. Next, in section Industry we explain how we expand previous work by^1,2^ for industry classification. The core of our work concerns a heuristic which assigns individuals to supervisory boards and management boards of firms as well as gender attribution. For each heuristic, we provide a verification of the validity. We first identify individuals as board members, which is described in section Managers. The heuristics of the assignment to the board are described in section Board assignment. The final stage consists of assigning the periods in which specified individuals held these positions. Our method to assign the dates of holding a given position is covered in section Timing the board members. Our heuristics for gender attribution are described in detail in section Gender attribution.

### Preprocessing orbis data

The aim of this section is to describe in greater detail the Orbis data and the pre-processing. Orbis unifies data from information providers from all over Europe. The information providers are individual contractors who collect data for Orbis in respective countries. They reach out to national official public bodies in charge of collecting annual financial statements from national registry records, courts, and firms. They provide Orbis with firm-level data consisting of balance sheets and profit-loss statements reported by the companies to registry courts and local government statistical offices. Some data is taken from end-year reports or scraped from the websites of these firms by Bureau van Dijk, which compiles the Orbis data. As noted by^4^, about 99% of the companies in the data set are private.

Orbis data used to be available in waves disseminated annually. Each wave of Orbis covers up to ten years of firms’ history for all the firms available in a given wave. Data availability varies across countries^5^. As of 2020, Orbis data are distributed as Orbis Historical Data, which, in addition to current 2020 data, provides partial information on historical records.

We concatenate 11 waves of Orbis database of Bureau van Dijk, namely: 2000, 2002, 2003, 2004, 2006, 2008, 2010, 2012, 2014, 2016 and the 2020 Orbis Historical Data (OHD). It is not clear which firms were included in the OHD. Using the unique firm identifiers of the Orbis data (the so-called *bvdid*) we compare the OHD sample with the compilation of the other 10 waves. In total, 27,649,381 unique firm identifiers matched between OHD and the combination of the other 10 waves, and 71,536,992 did not. Of the nonmatching identifiers, 6,147,066 unique identifiers were available in the other ten waves, but were not reported in OHD. By contrast, 65,389,926 unique identifiers were available in OHD and were not available in our ten waves. While it appears that OHD is broader, data availability is smaller in OHD. In fact, historical registry data covering individuals in executive and non-executive positions is much more sparse in OHD. We document these major differences in section Timing the board members.

#### Commonalities with previous work

Our own experience concurs with^1,2^ on all points related to exporting the individual level data through Orbis interface, as well as mapping and organizing the files in the OHD distribution. Specifically^2^, provide a step-by-step guide on how to obtain data from Orbis, as well as how to merge various data sets provided by the company.

Since^1^ worked with one wave of Orbis data, they did not address potential issues related to the overlap of ten-year windows. Recall that each annual wave of Orbis provides up to ten years of historical data. Consequently, data for a given financial year can be reported in more than one of the available waves. If the values are identical, this redundancy is immaterial. If the values are missing in one wave, but are available in another wave, we are able to lengthen the within-firm panel.

In the period covered by Orbis data, the industry classification changed several times. Our procedure of harmonizing the sectoral codes builds on the approach of^2^, who match the codes across classifications based on the content. We pursue similarly. A strength compared to^2^, who work with two-digit industries, is that we provide cross-walks for four-digit industry classification. We describe how we augment their approach in section Industry below.

### Legal form

Since the purpose of the database is to study gender board diversity, we select a setting in which this is meaningful. Therefore, for the construction of this database, we restrict it to firms with formalized boards. To identify such firms, we rely on national legal forms because the obligation to have a board may depend on specific criteria such as a specific type of business vehicle. For example, in some countries limited liability companies are obliged to have both executive and non-executive managers, whereas in others additional criteria may apply (such as annual turnover or headcount). Legal requirements for registration and reporting differ across countries covered by Orbis. Similarly, data collecting procedures differ. Bureau van Dijk does not collect data themselves. Bureau van Dijk delegates the data collection effort to forty different information providers including business registers. In Table A.6.1^2^, provide a registry of information providers across countries covered in Orbis. Consequently, in some countries, data is available for every business, whereas in other countries availability is restricted to some legal forms. As of 2010, Orbis provides a classification of firms into a standardized status: sole proprietorship, partnership, private limited company, public limited company, branch, etc., but this classification is insufficiently detailed for our objectives.

We deploy a variety of Internet sources, including OECD country reports on corporate governance standards, as well as tax advise classifications to classify firms into those which ought to have a management board only, and those that are legally bound to have non-executive (supervisory) board or board members as well^6^. provide a classification of European countries over time; there are three corporate governance systems

1. two-tier - clear distinction between two governance boards: management (executive) and supervisory (non-executive, independent), including the mixed system;
2. one-tier - management (executive) and supervisory (non-executive or independent) directors operate within one board;
3. other (including the audit board system, responsible for legal compliance, functioning alongside the management board, most common in Italy and Portugal).

In the mixed system, the executives take daily management decisions, reporting to the non-executive members during the general meeting (it is most common in Nordic countries, see^7^). In the remainder of this paper, we will use interchangeably terms management board members and executive directors, as well as supervisory board members and non-executive or independent directors.

We proceed in the following steps. First, we tabulate the national legal forms reported in each wave of Orbis. We do this separately for every country. Second, for each of the legal forms, we inquire if that type of entity is obliged to have a management board and/or a supervisory board, using the available literature on corporate governance. We started from a list of specific keywords in the legal form; for example, a limited liability partnership (LLP) in most legal systems must have a board. We thus classify the most common legal forms. Third, we reiterate for less common legal forms, until all forms have been classified. Fourth, we proceed systematically for every country. In these four steps, we establish a dictionary of firm types that indicates which ones are obliged to have at least a management board. We then tabulate the legal forms in Orbis for the unclassified firms, reiterating the entire procedure, until virtually all cases are classified. The classification is eventually translated into the algorithm, which permits replicating our procedure. Rather than working with lengthy tabulations, our algorithm effectively works with morphemes (parts of names) rather than with the full names of legal forms. Such morphemes include for example “limit”, “liab” and “partn” instead of relying on the full legal name “limited liability partnership”. Morphemes help us circumvent a large share of misspellings and random abbreviations. Working with morphemes makes our approach robust across countries with similar legal forms. The dictionary of morphemes is distributed as part of our codes and includes terms in English as well as in local languages. This is because Orbis often reports legal forms using country-specific abbreviations and names. Examples include SARL in Francophone countries, AG in Germanic countries, etc.

This procedure identifies firms whose legal form determines that they should have at least a management board. In addition to legal forms without an obligation to have a board (such as sole proprietorship), Orbis includes a number of non-commercial institutions which are out of scope for this analysis (such as cooperatives, charitable institutions, NGOs and even public institutions), which are also excluded from the sample based on our legal form classification. Overall, 64,656,554 firm-year observations concerning sole proprietorship as well as non-profit entities as well as 15,497,240 firm-year observations for cooperatives and private partnerships were excluded.

Our sample covers 79,186,373 unique firms, which at least in some years are obliged to have and report a board or boards. We have 326,824,617 firm-year observations who ought to report at least one board member, Which implies that on average we observe a firm for 4.13 years. In this sample, 9,932,642 (13%) report no people, and 69,253,731 (87%) report at least one person. In the remainder of this text, we explain how we provide coherent industry classifications for those firms, how we identify board members, and how to delineate between management (executive) and supervisory (non-executive) boards.

### Industry

The Orbis data report NACE classification at four digits. The classification is taken from and is valid for a given Orbis wave. For example, the NACE code of a firm available in the 2014 wave refers to industry reported by this company in that year, which is NACE Rev 2.0. Our data cover the years 1990–2019. During this period, the NACE classification has changed twice: Rev 1 was replaced by Rev 1.1 which in turn was followed by Rev 2.0.

The changes in NACE classification are not an issue for firms observed under both classifications. By combining many waves, we are also able to identify firms that switch their main sector of activity. However, the change in NACE classification constitutes a challenge for the firms that were observed under only one classification. Orbis does not provide historical NACE codes for firms, even when the historical data cover the period from the previous NACE classification. Thus, to maintain comparability across years, crosswalks need to be developed. The crosswalks are necessary only for the firms which appear in Orbis for the first time under newer classification, but its retrospective data cover periods of older classification. Similarly, we need to obtain consistent NACE codes for firms that existed only before a change in classification(s) and are not available in the newer samples.

Our procedure for developing crosswalks is consistent with^2^. Specifically^1^, describe their approach to NACE harmonization in Section A.5.2, p. 50 in the accompanying documentation document. However, we cannot rely on these codes for two reasons. First, we need four-digit classifications, whereas^2^ provide two-digit crosswalks. Further, our sample covers two changes in NACE classification, whereas^2^ cover the change from NACE Rev. 1.1 to NACE Rev. 2. Ultimately, our procedure assigns firm-year classifications according to NACE Rev. 2 for all periods.

Our procedure involves three steps. First, we identify the firms when Orbis reported the same firm in two or more classifications. In these cases, the firm itself reported its actual NACE of main activity. For the remaining firm-year observations, we need to provide crosswalks. Hence, in the second step, we apply all the unique crosswalks made available from the official correspondence tables distributed by Eurostat (the so-called RAMON database: https://showvoc.op.europa.eu/). Unique crosswalks cover roughly 54% codes and approximately 42% of firm-years. Third, for the cases where the correspondence tables are many-to-many, similar to^2^, we manually match the codes by reading the full descriptions of the codes. We review the area of firms activity under the reported classification and assign the missing NACE Rev 2 classification from among the relevant options^2^. use the top NACE code in a given two-digit group. We do the same for the four-digit codes. If a given activity is completely missing in the official correspondence tables, we read the content and try to find a matching classification. If no such code exists, we assign the most similar code not elsewhere classified. For example, NACE Rev 1.1. has a section 45.25 *Other construction work involving special trades*. No section with a similar name exists in NACE Rev 2. In this case, we assign 43.99 *Other specialized construction activities n.e.c*.. We proceed in a similar way in other problematic cases. Our own correspondence table is part of this documentation.

Note that for some firms, NACE classification provided by Orbis is at two or three digits rather than the full four digits. In those cases we assigned the adequate two or three digit in the older/newer classification(s). This step is identical to^2^ for two-digit codes and follows the same spirit as four-digit for three-digit codes.

For 2.77% observation no NACE information is available. In some cases, the fact that NACE data is missing can be rectified by imputation. Specifically, if a given firm has a NACE code reported for years prior to a missing observation, as well as for years after the missing information, we test if the two available NACE codes are identical. If that is the case, we fill in the missing information with this specific NACE. This way we are able to recover 454,762 firm-year observations (0.13% of the sample or 4.7 of the missing cases). Ultimately, we assign 97.24% of firm-year observations with a NACE Rev. 2 classification. The remaining 2.76% consists of two cases: no NACE was provided (2.64%) or we could not find a satisfactory crosswalk (0.12%).

### Managers

Orbis provides detailed information on the executives and non-executives of firms. This detailed information, dubbed DMC by Orbis (the name comes from “directors/managers/contacts”, which was the historical name of this part of the data). It comprises name, surname, function name, position (job title), level of responsibility, appointment date and resignation date. The *manager function* name is a concept distinct from the position or the level of responsibility in Orbis data. The *manager function* name is given either in local languages or in English. The function name is specific to a firm in a sense that across most–though not all–countries in Europe, firms are free to utilize any names for their functions. The position and the level of responsibility are variables defined by information providers or directly by the Bureau van Dijk, based on the function name. We discuss the differences between these two concepts in section Board assignment. The most recent waves of Orbis include data on gender, nationality and birth year of those individuals, but this crucial information is missing for most of our data. In this section, we describe how we process data on managers to arrive at board assignment as well as gender attribution.

First, we strip all records of diacritic signs characteristic of many languages. Second, we observe that the DMC database comprises legal persons in addition to physical persons. that is sometimes firms are reported instead of individuals. We exclude legal persons by identifying the strings associated with legal forms in the *name* variable. Based on the inquiry into the legal forms across firms, as covered in section Legal form, we construct a dictionary of legal form names. For example, in the case of Germanic countries, the dictionary includes KG (alternatively: *Kommandgesellshaft*), GmBH (*Gesellschaft mit beschraenkter Haftung*), etc. Likewise, in the case of Francophone countries, the dictionary includes SARL (*Societe a responsabilite limitee*). The full list of terms to identify legal persons is part of this documentation.

Third, in some cases the individuals listed are reported as shareholders or representatives of stakeholders, e.g., trade unions. In these instances, their names and surnames are missing and they are solely described by their function. Since individual details are unknown for such records, they also have to be removed from the sample.

Overall, although the DMC data is reserved for physical persons, occasionally it conceals legal persons. Whenever a record reports a name that we classify as a legal form or as a function rather than a physical person, it is dropped from the sample. This restriction is not arbitrary, as we follow the dictionary constructed in section Legal form. It is also quantitatively minor. In total, this procedure reduces the sample by 3% of all firm-year records.

Once we have a list of physical persons, we parse each record for name and surname. The *full name* variable includes salutations (Mr, Mrs, Ms), suffixes (for example: Jr.) and titles (for example: “Lord”, “Prof.”, “Dr.”, “Rev.”, etc.). We trim those elements from the *full name* variable. We also trim unnecessary spaces and signs. We parse the remainder for the given name and the surname (family name). This identification is necessary because we are assigning gender based on linguistic rules which apply to given names in some languages and to family names in other languages. We start by counting the remaining words in the *full name*. We inspect each country separately, and in most cases, we take the first word as the given name and the last word as the surname. In the case of several countries (Romania, Serbia, Spain and Italy), the family names are reported as first and the given names are reported as the last.

In total, we obtained 249,668,837 records. These records are identified at the level of person × firm × function × Orbis wave. We refer to this sample of managers in the remainder of this documentation. We also describe how we assign individuals to boards and how we assign dates of appointment. Note that these 249,668,837 records do not identify unique individuals: a given non-executive director can be a member of the Nominating Committee and an Audit Committee, in which case this person would show up twice at this stage. While these two positions describe responsibilities, both belong to a supervisory board and can be held simultaneously by one person. We describe the board assignment in the next section. Further, section Timing the board members describes how timing is assigned to board positions for each individual.

### Board assignment

**Fig. 4:**
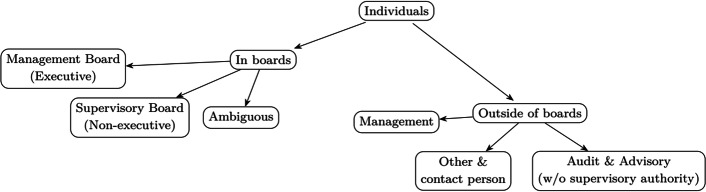
Categories of individuals reported in Orbis data.

We classify managers according to their primary function in the organization, as portrayed in Fig. 4. Given the legal and institutional complexity, as well as differentiation across countries covered by Orbis (see^6^), such an assignment is neither universal nor entirely obvious. Our aim is to draw a line between the day-to-day executive and management roles of top corporate governance members and non-executive, supervisory roles of oversight and control.

As discussed above, legal forms are informative about whether firms should have a management board, a supervisory board, or both or neither. We retain only those firms that are obliged to have at least one board. Typically, registries include information on individuals filling the positions in the required boards. One individual may have a management position in one company and a supervisory position in another company. Hence, all classifications must be applied to the appropriate sample of companies, and must be determined at the level of each record in our database, that is, company × person × position.

Orbis data provide two sources of information about the positions of respective managers. First, the database includes the *manager function* variable. This is a text field. This variable reports values in either local languages or in English. These strings may occasionally be misspelled, abbreviated or altered in some other way. The second source of information is the position assignment generated by Orbis, with two variables: *position* and *level of responsibility*. Among our Orbis waves, the first one to report this classification is 2010, and hence we could not rely on it for the earlier years in our sample. Furthermore, the Orbis classification rules have changed between waves and are not sufficient for the purpose of our research. This section characterizes classification by Orbis in the context of our research needs in section Amadeus classification to boards. Next, we describe our heuristics for assignment to boards, based primarily on the *function name* in section Our heuristics. We conclude by a comparison between our assignment and Orbis classification, where we provide the estimates of the overlap and explain the origins of differences in section Comparing our heuristic to Orbis classifications.

#### Amadeus classification to boards

Orbis provides two variables *level of responsibility* and *type of position*: “[w]e make manual matches by source to take into account the context of the country instead of general word parsing. The same original job title may end up in a different standardized position.” The classifications by Orbis are shorthands for full positions.

- *level of responsibility* - this variable typically delineates between a board-level position and other high-level executives. This variable takes on values such as “Founder”, “Member”, “Highest executive”, “Human Resource executive”, “Unspecified executive”.
- *type of position* - this variable typically refers to the area of work/expertise, such as “SupB”, “RiskC”, “SenMan”, “IT”, “Sales”.

These variables were not available before the 2010 wave. Furthermore, subsequent waves of Orbis data report different values/categories for this variable and there are no cross-walks provided within the data or its publicly available documentation that could guide us in harmonizing these values/categories between the years. Taking values/categories from all waves when they are available, We interpret the Orbis classification as follows in Table 2.

**Table 2:** Classification in Orbis data.

Category	Type of position	Level of responsibility
Management board	ExeB, ExeC, BrOff, SenMan	Chief, Executive committee, Executive Head
Supervisory board	SupB, AudC, CoGoC, RiskC, NomC, RemC, ChmC	
Ambiguous board	BoD, AdvC	Member, President, Chair, Representative, Board
Other	FinAcc, OthDep, LegalDep, Proxy, Proc, IT&IS, MarkAdv, Oper, Sales, HR, Qual, R&D, Oper, AdmDep, Health, CustSv, EthC, EnvC, SpecPos, CSRC, GovAff etc.	Highest Executive, Unspecified Executive, Marketing Executive, Other Department etc.

With this classification, we could pursue to identify members who belong to a board, but only in a limited number of cases we could delineate supervisory (non-executive) from management (executive) positions. Thus, we could potentially leverage Orbis classification, but we could not rely on it completely.

Although the two Orbis variables provide much information, they are also known to be internally inconsistent. As noticed by^8^ and^6^ Orbis classification is not always consistent with the rules of corporate governance. For example, Orbis assigns “Liquidator” an executive role even though it is not equivalent to a management board. We proceed to comparing our heuristic with Orbis classification in the few waves, where both are available. Note that for the waves prior to 2008, only our heuristic can be applied to classify individuals.

#### Our heuristics

Our heuristic is primarily based on the *manager function* variable. This is a text field, filled by the companies. The values of this variable are given in English or in local languages, with multiple abbreviations and variations. We begin by tabulating within countries. In some countries, there were only a few categories (e.g., Estonia), whereas in other countries the tabulation included more than 1,200 cases.

By browsing through those logs, we identified the keywords that were common to all countries. For example, English names such as variants of *CEO* or *non-executive director* were relatively common. In parallel, we identify multiple country-specific keywords. For example, the data for France include relatively frequently a variant of *PDG* (*President Directeur General*), data for Germanic countries frequently report *Vorstand*, whereas Spanish and Portuguese-based firms often include a variant of *Conselho Fiscal*.

Parsing the words, we establish the list of morphemes which are consistent with the non-executive (supervisory) board. This list includes term related to non-executive, independent, supervision, audit, nomination/compensation/appointments as well as the general assembly. Analogously, we generate a list of manager functions which are consistent with the executive (management) board. This list includes terms related to C-suite positions, executive, and a variety of specific areas of operations such as finance, HR, IT, treasury, or operations. The list of top management positions included all variations of CEO/PDG general director/manager across many legal forms. Working with morphemes of each part of the *manager function* value separately makes our heuristic effective irrespective of the ordering of the words (e.g., *general manager* vs *manager general*, which could depend on the specific language). Naturally, we also develop language-specific lists of morphemes. Note that working with morphemes helps to ignore some language specificity, because the morpheme “direct” encompasses both the English *director* and the French *directeur* as well as a *directrice* and many potential misspellings of these terms.

Our procedure was iterative. We applied our lists to the *manager function* and tabulated the unassigned values to update the list of morphemes, until no more positions could be satisfactorily attributed to the management board (executives) and supervisory board (non-executives) or at least to either of those boards (the ambiguous board assignment). The full list of morphemes in the form of a do-file is a part of this documentation. The iterative approach serves to identify all morphemes correctly and avoid misattributions.

Our heuristic treats all other *manager functions* as irrelevant in the sense that these are either contact persons for the Orbis information providers, or individuals in high management positions, but not the top echelons of the hierarchies. Note that our heuristics are country specific. For example, for some legal forms a statutory auditor is a formal function, involved in supervising the work of management, even if not in capacity to nominate or remove management board members (e.g. Italy and Portugal). Thus, these positions are semi-supervisory board in some countries. However, in most other countries, statutory auditors are similar to compliance officers: they are unable to make their own management decisions, and they lack any supervisory authority. We identify multiple such cases across the countries covered in Orbis, and our heuristics comprise many country-specific rules, in addition to general rules.

Ultimately, we were left with 49.6% of cases, where we could not assign individuals to any of the boards. Often this occurs because Orbis reports merely that an individual is a member of a board (of directors). Neither in the case of one-tier, nor in the case of two-tier systems, such description is sufficient to delineate between executive and non-executive positions. Estonia is a single exception from this ambiguity, because in this country, “Board Member” is a C-suite position whereas “Council Member” is a supervisory (non-executive) position. However, this description is sufficient to state that an individual holds either of the two positions. To accommodate those individuals, we create a third category, which encompasses ambiguous board assignments.

Since some individuals are unassigned based on the information provided directly by firms to the Bureau van Dijk information providers, we augment our heuristic to limit the scope for omissions. We take into account the information included in the Orbis-derived variables. In other words, whenever our heuristic delivers no assignment, we also apply our heuristics to categorizations provided by Orbis-derived in the variables *level of responsibility* and *type of position*. Using this second step of board position assignment serves two purposes. First, we can assign individuals whose position remains unclassified based on the *manager function* variable. Second, we verify if individuals × firms assigned as ambiguous could be potentially assigned to one of the boards with certainty. Note that this second step can only be applied to data as of the 2010 wave.

Table 3 reports the results of augmenting our heuristic with the heuristic based on Orbis-derived in the variables *level of responsibility* and *type of position*. We improve the board assignment by 7.4 percentage points, most of those individuals are assigned to a management board.

**Table 3:** Heuristics for board assignment.

Heuristic used	Management board	Supervisory board	Ambiguous board	Other
Manager function	84,506,960	2,934,868	38,428,865	123,798,144
(% of total)	33.8%	1.2%	15.4%	49.6%
+ additional variables	+ 12,582,944	0	+ 5,829,810	−18,412,754
All heuristics	97,089,904	2,934,868	44,258,675	105,385,390
(% of total)	38.9%	1.2%	17.7%	42.2%
*Notes:* This table reports the number of cases assigned to corporate bodies with our heuristic, based on *manager function* variable and augmented with variables *type of position* and *level of responsibility*, both of which are Amadeus-derived variables.				

#### Comparing our heuristic to Orbis classifications

Comparing our augmented heuristic to Orbis classification helps to establish two observations. First, we can observe the overlap between our heuristic and the Orbis classification. These values can be inferred from the diagonal of Table 4. Second, we can identify the main types of conflicts between our heuristic and Orbis classification. We discuss both these sets of observations below.

**Table 4:** Board assignment: comparing our heuristic to Orbis classification.

Orbis	Our augmented heuristic			
	Management board	Supervisory board	Ambiguous board	Other
Management board	**70,445,424**	140,855	225,563	63,299,072
	**90%**	6%	0%	72%
Supervisory board	4,942	**1,559,900**	74,143	29,707
	0%	**68%**	0%	0%
Ambiguous board	1,355,691	543,888	**31,629,140**	937,497
	2%	24%	**99%**	1%
Other	6,113,182	49,850	91,495	**2,427,253**
	8%	3%	0%	**27%**
*Notes:* This table reports the outcomes of tabulating our augmented heuristic on board assignment against a classification derived from Orbis data, in line with Table 2. The percentages reported add to a 100% in columns (up to rounding errors). The data concerns waves 2010 and subsequent ones, including OHD.				

##### Management board

We observe a high match in the management (executive) board assignment between our augmented heuristic and Orbis classification. Recall that augmenting our heuristic based on *manager function* name delivered roughly 7 percentage points higher share of identified cases. Meanwhile, over 90% of records for which both measures can be obtained receive the same assignment from both approaches. Additional 2% of records are management board according to our heuristic, but could not be assigned to a specific board according to Orbis classification. This is not surprising, our heuristic explores additional information in the variable *manager function*.

Similarly, we can explain the biggest discrepancy for the management board assignment, which comes from the fact that our heuristic delivers a direct assignment to the management board, whereas Orbis is dismissing these positions as unclassified. Of the circa 6 million such records, the vast majority is explained away by merely few obvious mis-attributions by Orbis.

**Table:** 

**Explaining the mis-attributions #1. Our heuristic: management. Orbis: ambiguous**.
• 823,838 Members of the Board in Estonia, which are unequivocally executive positions according to the corporate governance rules of this country;
• 166,507 members of the Administrative Board in Luxembourg, which are the management board positions according to the corporate governance rules in this country;
• 66,788 Members of the Administrative Council in Greece, which is a local name for the management board.
Taken together, these are 1,057,133 cases, roughly 78% of all discrepancies in this category.

**Table:** 

**Explaining the mis-attributions #2. Our heuristic: management. Orbis: other**.
• 2,066,208 cases concern the position of *general manager/director* and *executive director* in Francophone countries, Russia, UK, Spain as well as Denmark and Norway;
• 1,683,183 cases concern managing director and general partner in Germany and Austria;
• 383,931 cases are *executive head* in Czech Republic, it is the local language equivalent of general manager;
• likewise, Hungary’s local language equivalent is *Office-bearers*, which constitutes 165,468 cases;
• 260,875 cases in Portugal, in limited partnerships, *managers-partners* are management board positions;
• 192,883 cases in Poland the *president* can only be a management board position;
• in the UK, 121,833 cases of finance director, who are top-management
• Orbis considers 47,105 Chief Executive Officers in Switzerland to be non-board positions.
Taken together these are 4,798,930 cases, or roughly 78.5% of all discrepancies

For 225,563 cases we have mismatches where Orbis assigns the individual to be member of the management board, but based on our two-step heuristic, we can at best assign this person to a non-specific board. This mismatch is explained in more detail below, when we discuss how our heuristic might lead to ambiguous assignments.

##### Supervisory board

The raw match for the supervisory (non-executive) boards reaches over 68%. In addition to 1.5 million concurring cases, Orbis classifies 140,885 records as management board, whereas our heuristic assigns those cases to non-executive board. This discrepancy consists of solely three clear misattributions by Orbis described in the box below. Thus, our heuristic proves to provide a more reliable assignment.

Further discrepancy concerns 540 thousand records which are assigned no specific position in Orbis and who are members of supervisory boards according to our heuristic. Three cases of clear misattribution explain 380 thousand cases. For example, a generally ambiguous *board of directors* position can be only a non-executive position in Russia. Once these mistakes are corrected in Orbis, we total 92% of concurrent classifications.

**Table:** 

**Explaining the mis-attributions #3. Our heuristic: supervisory. Orbis: management**.
• 85,616 individuals × companies described as Member of Council in Estonia;
• 56,461 records who are literally described as supervisory board in Hungary in *manager function* name;
• 38,262 records described as independent directors in France.

**Table:** 

**Explaining the mis-attributions #4. Our heuristic: supervisory. Orbis: ambiguous**.
• 284,317 correspond to members of the board of directors in Russia;
• 77,113 cases for Members of the Council in Estonia, a position that unequivocally belongs to a supervisory board;
• 47,668 employee representatives in Norway and Denmark, which matches a supervisory board description;
• 28,760 members of general assembly or audit board in Portugal
• 13,552 non-executive directors in the UK

There are 74,143 where Orbis claims a supervisory board position, whereas our classification states ambiguous. This category is essentially explained by 67,222 advisors in AG and EG companies in Germany and Austria. The advisors to supervisory boards are not actually supervisory board members, so this is a case of mis-attribution. Finally, there are roughly 30 thousand cases that Orbis assigns to the supervisory board and our heuristic claims are not persons of interest, of which 28,783 cases are the position of censor in Romania. According to the corporate governance rules, this position has compliance responsibilities, but no decision-making authority.

Given that we list clear misattributions in Orbis classification, we judge that 98% of the supervisory board cases match between our heuristic and the Orbis classification. There could be concerns that there are fewer records within supervisory (non-executive) positions than in the management ones, but we are assured that our heuristic delivers a reliable classification into the management (executive) as well as supervisory (non-executive) boards. The majority of the discrepancies between our heuristic and Orbis classification can be explained away by clear cases of mis-attribution by Orbis. While Orbis classifications are not available up to 2010, the comparison between our heuristic and Orbis classification reveals that once available the Orbis assignment to management and supervisory boards should be taken with caution.

##### Ambiguous board

Our heuristic delivers approximately 32 million ambiguous board records. Our heuristic is 99% concordant with Orbis. These cases refer to individuals who are member of a board, but where we cannot determine whether this is a management or supervisory board. This problem can be attributed to the fact that the *manager function* description is incomplete. For example, partners and shareholders can be both in executive and non-executive positions. Likewise, *board member* or simply *BoD* (for board of directors) descriptions are ambiguous. Despite taking the two-step approach and utilizing information from two additional variables (i.e., *level of responsibility* and *type of position*, both of which are designed by Orbis), one cannot establish which board/position is the adequate one.

The discrepancies between our heuristics and Orbis classification are minor. There are about 100 cases in the UK, all of which concern chairmen, (vice)president and member of the LLP. Trivially, these three positions can be either management or supervisory boards, and Orbis provides no additional details that could help identification. However, Orbis classifies them as management board. Furthermore, one single group of advisors (in Luxembourg, Austria and Germany) explains the discrepancy of over 70,000 cases where our heuristic arrives at ambiguous board, but Orbis claims supervisory board without any additional information. Finally, nearly 60% of cases where we claim ambiguous and Orbis claims no board position concerns shareholders/partners in Greece and in Italy.

##### Other

The last column of Table 4 reports a total of roughly 64 million observation where our heuristic assigns no board, whereas Orbis classifies the cases into management board (roughly 63 million cases), supervisory board (nearly 30 thousand cases), and ambiguous board (roughly 937 thousand cases).

In fact, the three positions–unspecified *director*, unspecified *manager* and *company secretary* — constitute 37 million cases of the largest group, where Orbis assigns management classification and our heuristic implies a position outside boards. In some waves, Orbis classifies all positions described as *director* to the management board. Similarly, numerous positions across various countries are described merely as *manager*. Whenever the variable *manager function* signifies the top management position, our heuristic and Orbis classification overlap, but the 17 million cases have no additional information which would justify considering those individuals in those companies as c-suite. This, the misattributed case of director totals over 22.5 million medium-level directorship positions, which Orbis classifies as c-suite and our heuristic does not find grounds to assign either of the boards. In addition, Orbis classifies nearly *company secretaries* as executive board, whereas this position is neither management nor supervisory board per se. Similar to any higher-level management position, company secretaries may have the legal authority to sign contracts and they are in principle taking the responsibility of compliance officers. The company secretary is necessary to concur with many of the management board decisions, but in principle this is not an authority position in either management nor supervision of the management. They are not personally liable for company’s decisions (like the executive board) and they are not in position to change the management board of a company (like the non-executive boards). Thus, this is not a board position. Jointly, the company secretaries in the UK, Ireland an Cyprus explain away 14.5 million cases. The next biggest groups are administrators in Romania and Italy. Further, Orbis misattributes management to authorized officers in Switzerland. This position is akin to the operational director, it may be on the board (in which case it would be the management board, naturally), but it does not need to be. Finally, we identify in total in excess of 1,5 million liquidators (France, Germany, Hungary, Portugal, Romania, and Russia). While liquidators decisions are of paramount importance for companies undergoing bankruptcy, this is not a position elected by the shareholders to supervise management or appointed by the shareholders to engage in management. Liquidators are typically appointed by the court, based on availability and previous record in bankruptcies, rather than in management of successful businesses. Further, this category lists accountants and consultants. In neither of these cases were we in doubt about including those cases in any of the boards.

**Table:** 

**Explaining the misattributions #5. Our heuristic: other. Orbis: management**.
• nearly 17 million cases in the UK, most of which are medium level positions such as regional sales director, marketing director, etc. There are additional 3 million identical cases in Russia, and additional 1.3 million cases spread evenly between Belarus, Lithuania, Macedonia, Poland, Serbia, Slovenia, and Switzerland;
• nearly 13 million cases of company secretaries in the UK, 1,076,962 in Ireland, and nearly half a million in Cyprus;
• 3,715,433 cases of *administrator* in Romania and 1,228,027 cases in Italy;
• 515,485 cases of authorized officers in Switzerland;
• over 1.5 million liquidators in France, Germany, Hungary, Portugal, Romania and Russia
• nearly 1.2 million accountants in the UK and nearly 1 million consultants in the UK.

Our heuristic assigns management board position whenever the individual × company hinted in any way at board membership. In the absence of such indication in either *manager function* variable or in any of the Orbis-coded variables, there are no grounds to assign a board position. And indeed, a handful of quite obvious cases explains 44 million out of 64 million positions. The rest of the cases consist of either peculiar positions in the UK. Note that the UK is a special case, because it appears that throughout the subsequent waves the information providers reported the official occupation of the individuals, rather than specific positions within the company. We find more than 200 thousand cases each of teachers, farmers, nurses, electricians, architects, designers and housewives in the *manager function* variable, as well as over 600 thousand retired and over 300 thousand students. There are also many cases of positions which are clearly not c-suite level of management. Those include numerous cases of project managers, secretaries, consultants, engineers, doctors, drivers, lawyers, gardeners, etc. We do not report them in detail here, in the interest of brevity.

Of the 29,707 cases where our heuristic cannot attribute any board position, whereas Orbis classifies as supervisory board, 28,783 come from Romania and concern a single position: a censor. As we discussed, this position is similar to a company secretary in the UK, censors are expected to facilitate the compliance of management decisions with the legal system of Romania. While they cannot be fired by the management, they do not have the formal power to nominate the management or veto any decisions.

Surprisingly, the 937,497 cases classified by Orbis as an ambiguous board have a similar distribution of explanations as the management board, described above. Again, more than half of the cases consist of unspecified directors and company secretaries from the UK, Ireland, Russia and Cyprus. This parallel between the management board and the ambiguous board hints that Orbis classifications were not always applied consistently between countries and waves.

### Timing the board members

Once we assign individuals to a given position within each firm, we need to determine the periods for which that assignment is valid. In principle, for each individual, Orbis should report the date of appointment and the date of leaving the position, which is the date of resignation. However, Orbis methodology evolves across waves, as does the availability of this information. In fact, approximately 12–25% of board members (depending on the board and period) have complete information reported. We use two alternative information sources in Orbis to fill in these missing data.

For the first round of filling in the data, we use the confirmation date. This date is given by the information provider and indicates the date until which the information provided is validated before it is submitted to Orbis. This can be the date of the annual report or another document from which the information was collected. Consequently, there can be numerous subsequent confirmation dates for the same individual at the same company, in the same board or with changing boards. We assume that the latest confirmation date is the latest date at which we are certain the information was valid, likewise for the earliest. This attribution based on confirmation dates is obtained for a given person in a given board of a given firm.

In case of missing confirmation dates, we rely on the second source of information: for each wave, for each individual at each company, we should have an indication whether the information provided is current. Given that we have multiple waves of Orbis data, we can effectively leverage this information, and thus fill in the blanks in each of the years of our Orbis waves. Specifically, concatenating multiple waves of Orbis data raises the number of years for which we observe that “current” date.

In case of missing variables, a final method we applied is to use all the records for which some indication about the start of the appointment is available (e.g. year of appointment), but no indication as to the end is reported. This may be because information about the “current” date is missing. In those cases, we take a conservative stance and set the starting year to be also the ending year for each record; thus, a given person holds a position on a given board of a given firm for one year in case data on the ending of appointment is missing. We proceed analogously if the ending date is determined from appointment, confirmation, or “current” date, but no starting date is provided by Orbis. In other words, should only the ending or beginning be available, we assign a tenure of one year (with the starting date being equal to the ending date).

We utilize this additional information to complement the appointment dates with the two alternative sources of information in Orbis to determine whether a given person was a member of a given board in a given firm each year. We pursue in the following order. First, we assign dates using the appointment and resignation dates. Second, for all individuals who have the appointment date but have no resignation date, we inquire about the maximum of the available confirmation dates. If this information is unavailable, we take the maximum year for which the information is reported as current. If this information is missing, we assign the last year available for that firm. Third, in case appointment dates are missing, we take the earliest confirmation date. In case it is missing, we take the first available “current” year. If this information is missing, we take the first year in which a firm is observed in this legal form.

In fact, given the dates of appointment and the date current to a given Orbis wave, we are able to establish the duration of holding the position. For individuals for whom the date of appointment is available, this identification is sufficient. For individuals for whom the appointment date is unavailable, this identification rests upon the assumption that in the gap years (between the available Orbis waves), the individual did not leave the company to return later on. Note that for a current position, the resignation date is a future date at which the contract expires. This helps to fill in the gaps as well.

Note that in order to do the latest or the earliest of confirmation dates or “current” years, we need to define unique identifiers for managers. This identification cannot account for a variety of issues related to string fields: names can be abbreviated, or misspelled, the order of first and second name or surname can be altered, etc. Given the vastness of our sample size, we cannot fix those issues, which implies that average tenure is likely biased downward. However, this bias should be similar for both men and women.

In Table 5, we report the share of the manager × board × year observations. We organize the table in the order of applying the criteria to the data. For example, we only use the confirmation dates if year or appointment and/or year of resignation are missing. Each cell in this table reports the percentage of observations that have the time assigned based on a given combination of start and end criteria. For example 24% of supervisory board members in each year in a given firm have the complete dates assigned based on year of appointment and year of resignation.The percentage climbs up to 26% if we impute a resignation date based on the latest available confirmation date. We reach up to 93% of observations if we apply all the available criteria to the start and end of the appointment. The second number in each cell reports the average tenure in a given appointment. Continuing with the same example, for the most restrictive and least restrictive criteria, we obtain 4.86 and 3.6 years, respectively.

**Table 5:** Timing of board membership.

Information used for the ending → Information used for the beginning ↓	Year of resignation		+ the latest confirmation date		+ the last “current” year		+ the end year	
	Supervisory boards							
	%	# years	%	# years	%	# years	%	# years
Year of appointment	24%	4.86	26%	4.93	40%	4.94	43%	4.61
+ the earliest confirmation date	25%	4.70	30%	4.50	44%	4.65	48%	4.37
+ the first “current” year	26%	4.73	31%	4.58	87%	3.79	90%	3.69
+ the start year	27%	4.59	32%	4.47	93%	3.60	93%	3.60
	**Management boards**							
Year of appointment	12%	6.07	18%	6.47	36%	6.09	41%	5.43
+ the earliest confirmation date	14%	5.48	29%	4.86	46%	5.19	51%	4.76
+ the first “current” year	15%	5.71	29%	5.27	82%	4.38	88%	4.19
+ the start year	26%	5.21	31%	5.30	87%	4.19	90%	4.19
	**Ambiguous boards**							
Year of appointment	12%	7.03	20%	7.68	44%	7.36	55%	6.10
+ the earliest confirmation date	13%	6.58	22%	7.19	46%	7.15	57%	5.98
+ the first “current” year	14%	6.64	23%	7.42	82%	5.59	86%	5.41
+ the start year	15%	6.26	24%	7.15	86%	5.41	93%	5.07
*Note:* % denotes a ratio between observations falling in a given category and total number of observations; # years denotes the average of the tenure for observations in the numerator of the ratio in a given cell.								

Our approach proves to be conservative. While we expand the coverage of the sample with additional time assignment criteria, the average tenure systematically declines. If we were excessive in assigning time to boards members, the tenure would be increasing as more precise criteria are missing and less precise criteria are applied.

**Fig. 5:**
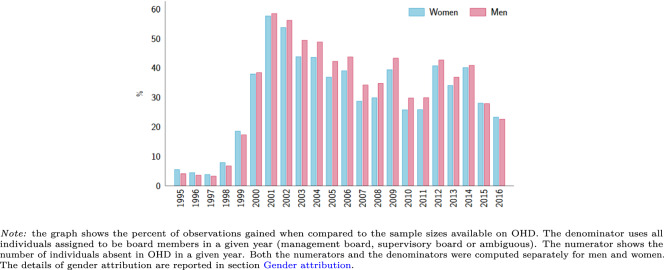
Percent of observations gained relative to OHD.

Once the time is assigned to every record in our database, we can meaningfully compare data availability between OHD and the combined waves of Orbis data. This is reported in Fig. 5, portraying the paramount gains from combining subsequent waves of Orbis in terms of additional data. Indeed, we have up to 50–60% more individuals in some years (both men and women) by combining subsequent waves when compared to OHD. This gain in sample size is due to two factors. As discussed earlier, some companies are reported in Orbis waves but are not reported in OHD, thus managers associated with those firms are absent in OHD as well. Quantitatively more relevant is the fact that the availability of the timing data is vastly improved from pooling Orbis waves.

For confirmation, we picked a number of well-known firms, for whom we could easily identify board members at given point in time. First, we collect the data about board members from the internet. Then we cross-reference names, surnames and board assignments in our final sample. While this is not a systematic validation of our sample, we can observe its strengths and the weaknesses. For example, for Volkswagen we confirm the same names for all five management board members in the 2008 wave of Orbis. We likewise confirm eleven out of 21 supervisory board members. In total, fifteen out of 26 individuals from the annual investor report for 2008 are recovered from the 2008 Orbis wave. However, after we apply our timing procedures, our heuristics deliver two management board members, two supervisory board members, and nineteen ambiguous board members. This is because the variables utilized in timing heuristics were missing in the Orbis data. We run a similar experiment using the 2010 and 2012 waves of Orbis, we report the results in Table 6.

**Table 6:** Identifying board members: a sanity check using the case of Volkswagen.

Year	Board	Official annual reports		Orbis wave		Orbis full sample		
		management	supervisory	management	supervisory	management	supervisory	ambiguous
2008		5	21	5	10	2	2	19
2010		8	24	8	17	5	11	10
2012		9	24	9	24	6	13	11

Three conclusions emerge from this example. First, while Orbis is very accurate in collecting the names of the individuals, the quality of both functional and time assignment were lagging in early years. Indeed, all three waves report all the relevant individuals, but fall short on giving sufficient information on when these individuals held positions at firms and which board it was. Second, gradually Orbis data is able to adequately match a high share of all board members and an increasing share of board assignment. Indeed, by 2012, our timing assignment becomes sufficiently accurate to identify adequately more than half of specific boards, and all individuals had adequate year assignment. Third, the case of Volkswagen illustrates that the issues with data quality are not driven by data availability. Indeed, all information for this company was widely available at the time. Either quality of data collection was insufficient or data processing between information providers and Orbis interfered to make the data incomplete in the distributed sample.

### Gender attribution

Only the 2008 and 2020 editions of Amadeus include a gender variable, and even then there is a significant share of individuals for whom this information is missing. These shortcomings strongly restrict analysis of gender and gender diversity on boards. To address this problem we propose a novel approach to gender attribution: we use names and surnames of each individual board member and linguistic rules to assign gender based on names. For most of the languages, full names, and surnames of individuals are sufficient to attribute a gender. We utilize a combination of heuristics to recover gender from names.

In the case of some languages, gender is directly identifiable from the form of the given name or the surname. For example, in some languages, surnames end with a gender-specific suffix (Slovak, Czech, Russian). In other languages the given names of women end with a vowel (Lithuanian, Russian, Slovenian, Polish). The procedure to distinguish names and surnames is described in section Managers. In Bosnia and Herzegovina, there is a comprehensive rule that certain vowels as last letters in a name identify women, hence lack thereof identifies men. The difference between these two rules is that the rule in Bosnia and Herzegovina is comprehensive, whereas the rule in Lithuanian, Russian, Slovenian and Polish the rule helps to identify women, but the lack of a final “a” is not a sufficient condition to identify men in these countries. The complete list of such rules has been compiled based on the World Atlas of Language Structures (https://wals.info with additional insights on https://en.wal.unesco.org/world-atlas-languages) and Wikipedia entries for each of the languages in countries covered by Orbis sample. The rules are decisive for Bulgaria, Czech Republic, Iceland, Lithuania, Macedonia, Poland, Russia, Serbia, Slovakia, Slovenia, and Ukraine. These structural rules constitute our first heuristic.

Linguistic and cultural scholars developed lists with books of names attributed to men and women. For example, there are no women named John in English, just as there are no men named Catherine. We utilize the most comprehensive source, the World Gender Names Dictionary (WGND^9^). We apply WGND to those individuals, whose gender cannot be determined using our first heuristic. To be able to match our names to WGND, we remove the diacritic signs. WGND is applied within each country, because the same name can be more typically male in one language and female in another (e.g. Andrea is more likely a woman in German and a man in Italian). This is our second heuristic. Note that the book of names is applied within a country and not within a language. For example, multi-lingual countries have names from all the relevant languages in their book of names. These books are missing, however, names of ethnic minorities or expats.

The two heuristics were applied sequentially: we use the first heuristic (based on the linguistic rules), then we proceed to WGND. Finally, some individuals bear names from a different country. We thus apply the entire WGND, without splitting the sample by country.

Conflicts may arise when gender attribution based on our heuristics and that based on WGND non-country specific attribution do not match. For instance an expat whose name ends with a vowel “a”, who works in a firm in Central Europe, will have a female gender in many of the Central European languages. This person would thus be attributed a female gender based on our heuristic even though based on global WGND that name could be classified as male.

Such conflicts can be resolved with the use of the salutation variable in Orbis. The salutations are reported from wave 2008 onward. We parse salutations to find most common words determining gender (e.g. Mr or Herr for men and Miss, Mrs or Frau for women). We do that for each language separately. Once we have a dictionary of male and female salutations, we can apply them to resolve conflict cases.

There are some cases in which gender identification is controversial or not possible at all. Sometimes individuals report incomplete or more than one name, which yields contradicting gender attribution (e.g. Jean-Marie is identifiable to a man, Jeanne-Marie to a woman, but J-Marie cannot be unequivocally attributed to any of the genders). However, in most countries, there was a small proportion of cases of conflicting gender attributions or missing gender attributions after applying all heuristics (around 5% of cases). Finally, the data for the Netherlands report only initials for names, whereas surnames are insufficiently informative about gender in Dutch. Hence, no gender identification is possible in the Netherlands, and this country is dropped from the analysis.

Table 7 reports the outcomes of our heuristics, with and without the use of salutations to resolve conflicts. We compare the gender attributions based on linguistic rules and names with the gender attribution provided by Orbis. Note that Orbis does not provide information on gender of individuals until 2020, with the exception of 2008 edition, so we could not rely on this variable in our research. However, its availability is helpful in determining the reliability of our gender heuristics. The table shows our attributions in columns and original Orbis attributions in rows.

**Table 7:** Gender attribution: comparing our heuristic and Orbis classification, the wave 2008.

Orbis	Our attribution						Total
	without salutations			with salutations			
	men	women	unattributed	men	women	unattributed	
men	1,652,300	7,306	88,738	1,657,524	7,358	83,462	1,748,344
	33.81%	0.66%	20.71%	33.88%	0.66%	19.82%	27.19%
women	2,793	273,569	26,026	2,833	274,564	24,991	302,388
	0.06%	24.55%	6.07%	0.06%	24.6%	5.94%	4.7%
unattributed	3,231,858	833,633	313,669	3,232,548	834,020	312,592	4,379,160
	66.13%	74.8%	73.21%	66.07%	74.74%	74.24%	68.11%
Total	4,886,951	1,114,508	428,433	4,892,905	1,115,942	421,045	6,429,892
*Note*: data come from 2008 wave of Orbis data. Only individuals who belong to supervisory board, management board or ambiguous board are reported in this table. Percentages add up to 100% in columns.							

It is immediately clear from Table 7 that our heuristic provides remarkably higher share of attributed individuals than Orbis. Indeed, of 6,429,892 records in 2008 wave, 4,379,160 remain unattributed in the original Orbis data. Meanwhile, our heuristic produces only 428,433 records without gender attribution (421,045 if we use salutations). Furthermore, while Orbis data recover merely 302,388 women (or 4. 7%), our heuristics attribute female gender to 1,114,508 cases (slightly more if we use salutations), which brings the women’s share on boards to 17.3%, nearly four times more. We can also observe that the majority of unattributed cases are not driven by conflicts in heuristics but by missing information. Indeed, salutations increase the share of attributed cases by a negligible percentage. Table 7 reveals as well that majority of mismatch between Orbis classification and our heuristics is driven by the unattributed cases in Orbis rather than by differences in the genders attributed. There is merely 7,306 cases (0.66% of all observations attributed to women), where Orbis claims male attribution and our heuristics suggest a female one. Likewise, there is merely 2,793 (0.06% of all observations attributed to men) cases when our heuristics deliver male attribution and Orbis reports female gender.

Overall, our gender attribution heuristic delivers great gains relative to the original gender reporting by Orbis. Not only are we able to attribute gender to a larger number of individuals, but also we recover a much higher share of women than would have been identified based solely on Orbis classification. When information is provided in Orbis, we identify no quantitatively meaningful differences in gender attribution by our heuristics and in the original Orbis variables.

### Final sample

#### Additional variables

##### Listed companies

Orbis data directly identifies publicly listed companies. This is of great value, as in many countries the legal form does not suffice to recover this status. This occurs because stock-based limited liability companies include firms that are not stock-listed companies, i.e. the stocks can be privately traded.

##### Ownership

Orbis provides ownership data for companies. This permits recovering networks of firms. The data comprises direct ownership (firm A holds % of firm B) as well as indirect ownership (firm A holds % of firm B, which in turn owns firm C and D). Orbis provides a sum of these percentages and based on these percentages classifies into groups such as majority ownership (above 50%), joint ownership (*n* owners of 1/*n*% each), total ownership (foreign company or branch). Our procedures are in line with Appendix B of^2^.

#### Descriptive statistics

Table 8 reports the final sample from the Orbis data. After adjusting the sample, we obtain nearly 30 million unique firms, and nearly 150 million firm-year observations, or roughly five years of observations on average per firm. Our sample contains information about nearly 60 million individual top executives and members of the supervisory board. We have slightly more than four person-year observations, or 243 million. Of these person-year observations, roughly 150 million are assigned to management (executive) positions. We identify nearly 4.5 million supervisory board (non-executive) positions. In addition, nearly 90 million positions are ambiguous, that is, we can ascertain that these are either management board or supervisory board, but not which one of those two. Finally, in our sample of 60 million individuals, roughly 44.7 million are men and 14.7 million are women, that is 24%. This is a very crude measure, and it does not take into account the time, cross-country, and cross-sectoral variation, we address those issues in the next section.

**Table 8:** Sample descriptives.

	# of unique obs.	# of obs.	# of observations in		
			management	supervisory	ambiguous
Firms	28,140,575	147,923,404	110,209,358	1,680,700	49,724,960
Listed	22,205	177,287	89,121	47,151	132,378
People	58,921,483	239,151,923	148,201,928	4,474,353	86,585,070
Men	43,675,552	180,887,653	113,260,406	3,428,028	64,288,678
Women	14,363,672	55,953,919	33,801,047	960,970	21,211,696
Total attributed	58,039,224	236,841,572	147,061,453	4,388,998	85,500,374
Women % in total attributed	24.75	23.63	22.98	21.89	24.81
Unattributed	882,259	2,310,351	1,140,475	85,355	1,084,696
*Notes*: The total number of observations is lower than the sum of observations for management board, supervisory board and ambiguous board assignment because for some firms there are two or more board assignments.					

**Fig. 6:**
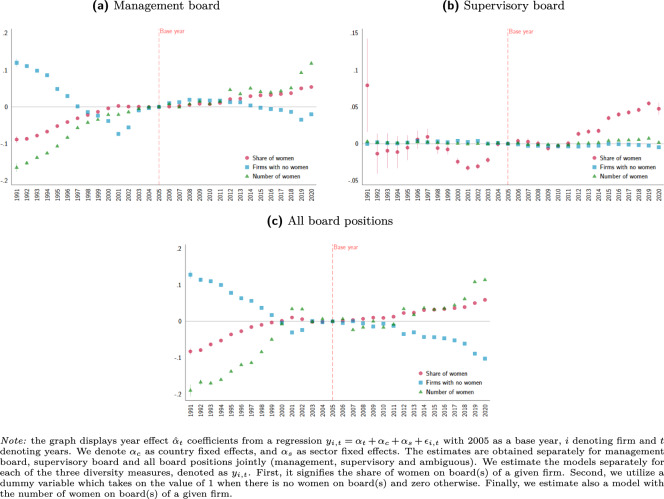
Time trends on measures of gender board diversity.

In Fig. 6 we present time trends in the three measures of gender board diversity, obtained with our data. The trends in the data appear to be very consistent for executive and ambiguous measures: important changes in early 1990s, stagnation in 2000s and picking up in the pace of change in 2010s. The supervisory (non-executive) positions display less clear patterns, with essentially no trends (except for the share measure).

In Table 9 we provide an analysis of variance, decomposing the variation in gender board diversity measures to country-specific factors, industry-specific factors and time trends. We show that most of variation is actually firm-level. Indeed, there is almost no valuable variation at the sectoral level. The data refute the concept of female or male industries. More variation–up to 14%–can be attributed to cross-country differences.

**Table 9:** The contribution of year, country and industry to variation in gender diversity measures.

Boards:	Management board	Supervisory board	Ambiguous board
Share of variation explained by:	Panel A: diversity measured as number of women		
year	0.3%	0.06%	0.1%
country	7.7%	1.3%	13.6%
industry	2.3%	0.2%	0.6%
Share of variation explained by:	Panel B: diversity measured as a share of women in sample		
year	0.2%	0.6%	0.2%
country	1.5%	3.4%	1.8%
industry	3.8%	1.3%	2.3%
Share of variation explained by:	Panel C: diversity measured as share of firms with no women		
year	0.1%	0.04%	0.2%
country	2%	0.7%	3%
industry	0.9%	0.08%	0.4%
total sample size	147,061,453	4,388,998	85,500,374
*Notes*: The table reports results of an ANOVA decomposition. We display the explained sum of squares in each cell divided by the total sum of squares for a given column in a given panel. Our sample has 36 years, 42 countries and 88 industries. Total sample sizes are reported in each column.			

#### Limitations of our approach

##### Individuals vs managers

Due to the frequent occurrence of misspellings even in the company name or legal form, we do not identify unique individuals in the entire sample. Note that adding a second name, abbreviating the first name, or misspelling would result in the same individual being treated as different people. Such alterations can occur not only within an individual’s history within one company, but also as they move from one company to another. Furthermore, if one individual holds positions across various companies within one capital group, it could be possible to observe some cases of misspelling and abbreviations. Less likely but nevertheless possible are the cases of two individuals with identical given and family name, who are two separate individuals, in the same company or in different ones.

##### Representativeness

Orbis data are not intended as representative. The process of collecting the data relies on information providers, who access public records available in a given country and obtain/digitize the available information. This information typically contains all registration data, so our data may be informative about the universe of registrations. However, reporting obligations vary between countries and vary over time. Thus, Orbis data are fairly indicative of the universe of firms in terms of registration data. Given that no record of “all firms” is publicly available for the countries covered by Orbis, it is not feasible to put this conjecture to an empirical test. Consequently, we are unable to judge what fraction of firms is dropped from the sample due to missing information about managers, their board assignment, time assignment or gender attribution. Once public registries of firms become available, testing the match between Orbis and public records is desirable, especially for the early years in Orbis sample.

In addition, for some types of companies, information providers digitize public records covering balance sheets and profit-loss statements. Given the differing reporting obligations, the reliance on financial records needs to be inspected for each study independently, see^2^ who discuss the coverage in terms of employment and value added.

## Data Records

The data is available in Harvard Dataverse repository^10^. The GBDD includes two files: a sectoral file and country file. The sectoral file includes indicators for -country-, -year- and 2-digit NACE code -nace2-. In each of those cells, we report the following variables.

- share of women in senior management -female_share_senmen- computed as an average share share of women holding senior management (executive) positions across firms; this is an unweighted share from all firms
- share of women in supervisory boards -female_share_supboard-, computed analogously for positions in supervisory (non-executive) boards
- share of women in all boards combined -female_share_boards- computed analogously; note that individuals in ambiguous positions are included as well
- share of women in senior management -female_share_weight_senmen- computed as a share of women holding senior management (executive) positions over the total number of individuals with such positions; this is a weighted share
- share of women in supervisory boards -female_share_weight_supboard-, computed analogously for positions in supervisory (non-executive) boards
- share of women in all boards combined -female_share_weight_boards- computed analogously; note that individuals in ambiguous positions are included as well
- share of firms without women in management (executive) positions -zero_share_senmen-
- share of firms without women in supervisory (non-executive) positions -zero_share_supboard-
- share of firms without women in any board -zero_share_boards-

Analogously, in the country file we include indicators for -country-, and -year-. The data is reported as an aggregate across all types of firms and separately for stock-listed firms and private (not listed) ones.

Figures 1–3 along with Table 8 report on the properties of our sectoral data. Our data covers roughly 60 thousand observations for most measures and roughly 30 thousand observations for measures concerning specifically the supervisory boards. The mean share of women averaged across firms, and averaged within an industry range between 10% and nearly 30%. The share of industries, where no firm has a single woman in the board, average 74% for the management board and 63% for the supervisory board positions. In the country-level data, we show that the mean share of women averaged across firms ranges between 10% and 30% for the private firms and between 5% and 25% for the stock-listed firms.

Given the massive size of our samples, our distributions are relatively smooth. Nonetheless, we see several spikes: at 0%, at 50% and at 100%. The distribution of the share of firms with no women is a less smooth, because it depends on the number of firms in a given four-digit industry rather than a number of board members and a number of women among them. The spikes at 0% for the measures of presence and at 100% for the measures of absence the fact that in relatively many firms and sectors, there are no women among the board members.

## Technical Validation

After adjusting the sample, we obtain nearly 30 million unique firms, and nearly 150 million firm-year observations, or roughly five years of observations on average per firm. Note that even the comparison with^3^, who also use Orbis data, is favorable. They end up with a sample of 4,7 million firms in total, with only one yearly observation for each. Their data on financial records is much more sparse, reducing their estimation samples to 2 million observations.

We validate our data in three ways. First, we do a sanity check for one, well-known company, handcollecting data for this company from alternative sources. Second, we compare the properties of our data to otherwise available samples. This is possible only for the listed companies because no alternatives to our data exist for non-listed companies. Third, we offer a systematic attempt to evaluate the consequences of our data harmonization decisions on identifying women among board members.

### Sanity check, the case of Volkswagen

For confirmation, we picked a number of well-known firms, for whom we could easily identify board members at given point in time. First, we collect the data about board members from the internet. Then we cross-reference names, surnames and board assignments in our final sample. While this is not a systematic validation of our sample, we can observe its strengths and the weaknesses. For example, for Volkswagen we confirm the same names for all five management board members in the 2008 wave of Orbis. We likewise confirm eleven out of 21 supervisory board members. In total, fifteen out of 26 individuals from the annual investor report for 2008 are recovered from the 2008 Orbis wave. However, after we apply our timing procedures, our heuristics deliver two management board members, two supervisory board members, and nineteen ambiguous board members. This is because the variables utilized in timing heuristics were missing in the Orbis data. We run a similar experiment using the 2010 and 2012 waves of Orbis, we report the results in Table 6.

### Comparing our data to BoardEx

Within this sample, 22,000 firms report being listed on at least one stock exchange, with nearly 180,000 firm-year observations. For comparison^11^, use ExecuComp data with 13,491 observations, whereas BoardEx data (used by^12^) covers 24,000 firms from European stock exchanges, the US, Canada, and other advanced economies.

**Fig. 7:**
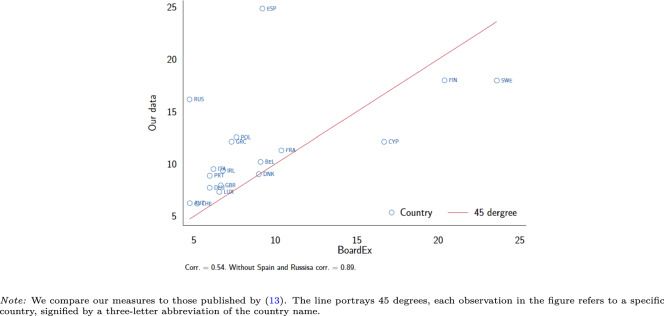
Time trends on measures of gender board diversity.

We compare our measures obtained from Orbis for stock-listed companies with analogous indicators from BoardEx^13^. provide average share of women in all board positions in 2010 within stock-listed companies. We compute analogous measures in our data and provide a country-by-country direct comparison in Fig. 7. Our data exhibit very high correlation with BoardEx-derived measures. There are two outlier countries: Spain and Russia. Especially for Russia, data coverage in BoardEx has been notoriously low, which may signify that a discrepancy between GBDD and BoardEx may be uninformative. Without these two countries, the correlation between the two data sources is 0.89.

### Processing of the data and the probability of identifying women in the sample

We explain three important methodological choices in our treatment of the data. First, assigning individuals to specific boards requires a case-by-case classification of positions in various legal systems. Second, we provide our own heuristic for timing of each person to each position. Finally, some firms have to be excluded from the sample due to missing information about sector. Although we cannot rely on Orbis own classifications, for several reasons–some concerns could be raised if our approach is perhaps overly restrictive and unintentionally eliminates too many women.

We address this concern by estimating a simple model. The left-hand side variable (LHS) takes on the value of 1 if a given person in our data is a woman and zero otherwise. With this definition, all unattributed individuals are in the baseline category of zero for the LHS. Thus, all estimated coefficients can be interpreted as measures of how conducive given methodological choice is to including women in the sample. A negative coefficient implies that a given methodological choice implies a lower probability of finding women in our sample. Conversely, a positive coefficient implies that, due to this modeling decision, we identify more women than in the baseline case.

The model is estimated at an individual-level. For each person in our sample, we construct a unique identifier. Note that this identifier is susceptible to misspelling of the first and/or second name, we discuss this issue in the next section. We then collapse all observations for a given person into a single record in each firm. If a person holds positions in many firms, we keep multiple records. For each firm-person record, we obtain measures synthesizing our modeling choices on the right-hand side of the estimation (RHS), as described below.

First, we test if listed firms are similar to non-listed firms. The dummy variable takes on the value of 1 when a given firm is listed, and zero otherwise.

Second, we check if firms that do not provide NACE classification are less likely to report women on boards. We drop firms which do not provide a NACE classification. One potential concern would be that firms with women among management and supervisory boards are less likely to be scrutinized by Orbis information providers. We construct a variable taking on the value of 1 if a firm provides NACE classification at all, and if the classification can be expressed in terms of Rev 2.0. Otherwise, the variable takes on the value of 0.

Third, we check if including the focus on management boards and supervisory boards eliminates women. We construct a dummy variable which takes on the value of 1 if a person has ever been attributed to a management board or a supervisory board and 0 if a person was attributed the classification of an ambiguous board. This measure is conservative in a sense that we try to verify if perhaps women are more likely to occupy positions that never get attributed to management or supervisory boards.

Fourth, we study the role of timing of board membership. OHD provides data on year of appointment and year of resignation. However, this data is not available for data which come from earlier waves of Orbis data; as documented in Fig. 5 we obtain many more observations by applying our timing heuristics. We construct two dummy variables. The first variable takes the most restrictive heuristic. This variable takes on the value of 1 if the timing is assigned based on year of appointment; and zero otherwise. This is akin to the first row in each panel of Table 5. Next, we also define a dummy variable that takes on the value of 1 if the timing is assigned based on year of appointment and year of resignation. This second timing variable is a subset of the first one. Thus, it measures the additional effect of using the restrictive definition of the year of resignation, on top of the restrictive definition of the year of appointment. These two variables help answer the question of whether our timing heuristics raise the likelihood of identifying women on boards.

The results are reported in Table 10. We find that firms that listed firms are less likely to have women on boards. Thus, expanding beyond listed firms, we actually raise disproportionately the probability of identifying women in Orbis. The effect is large, with the coefficient of roughly 7.4 percentage points or 30% of the average. Furthermore, we report that the NACE classification report fewer women than firms that do not report the NACE classification. Thus, excluding firms without NACE classification does not unintentionally eliminate women in greater proportion than men.

**Table 10:** Data processing and identifying women in Orbis sample.

	OLS	OLS with firm FE	OLS with firm FE & clustered SE
	(1)	(2)	(3)
Listed	−0.0743***	−0.0419***	−0.0419***
*(base level: not listed)*	(−40.00)	(−21.11)	(−21.03)
NACE	−0.0131***	−0.0171***	−0.0171***
*(base level: no NACE classification)*	(−119.49)	(−143.48)	(−128.83)
Management board or supervisory board	0.00161***	−0.00793***	−0.00793***
*(base level: ambiguous board)*	(13.31)	(−60.51)	(−49.87)
Timing relies on appointment date	0.0171***	0.0185***	0.0185***
*(base level: any other start date)*	(141.87)	(139.62)	(124.11)
and resignation date	−0.0167***	−0.0128***	−0.0128***
(base level: all other end dates)	(−93.20)	(−62.92)	(−53.38)
# of individuals in firms	63,852,221	63,852,221	63,852,221
Notes: linear probability model, constant included, not reported; t-statistic in parentheses. The sample in this regression is larger than 58,039,224 reported in Table 8, because we also include individuals who are affiliated with the firms for which NACE classification cannot be recovered. The symbols *, ** and *** denote *p* < 0.1, *p* < 0.05, and *p* < 0.01 respectively.			

Further, we find that women appear more frequently among management or supervisory board than among the ambiguous board. However, once we adjust for firm fixed effects, we find that, in fact, women are less likely among management or supervisory board than in the ambiguous board. In other words, if within a firm we find positions both attributed and ambiguous, then women are slightly less likely to hold an attributed position. The effect is not large: probability changes by roughly 0.8 percentage points, which is akin to 3% of the baseline probability.

Finally, we study the role of timing heuristics. In all specifications, the restrictive heuristics are more likely to identify women than the comprehensive ones. The appointment date appears quantitatively more relevant than the resignation date (1.7 percentage points vs 0.1 percentage points, respectively). However, both work in the same direction. Utilizing comprehensive heuristics, we obtain substantially larger samples but fewer women in relative terms. These two estimates reveal that more restrictive samples are likely to overstate gender diversity in Orbis data.

## Usage Notes

GBDD is helpful in addressing a wide range of research questions related to gender board diversity. We provide the data aggregated to the levels of each country in each year. This opens avenues for comparative research on institutional correlates (or determinants) of gender board diversity. Likewise, the consequences of changes to gender board diversity can be explored. Potential research questions include links to political participation, labor market equality, educational choices, etc. For example^14^, compare their hand-collected measures of gender board diversity among stock-listed firms with the indicators of gender equality across various domains in the United Nations Human Development Report 2006. With our data, one can expand the studied period to four decades and thus adjust the estimates for country fixed effects.

We also provide data disaggregated by sector level. Therefore, potential authors can combine our data with sources on wages and wage inequality, employment, productivity, TFP, and other indicators that can be obtained from alternative sources across sectors. For example^15^, study if greater gender board diversity reduces gender wage gap. They utilize cross-sectional data for the UK. With GBDD, interested authors can expand to a wide range of countries and obtain estimates that adjust for country specificity, sector specificity, or both. Analogous opportunities arise for the study of job demand, job stability, etc.

While the above examples are not an exhaustive list of potential uses of the GBDD, they demonstrate potential versatility and broad range of uses.

## Data Availability

For all users interested to work themselves with the Orbis data and verify or expand on our results, we distribute the full replication package. The codes which begin with raw Orbis data and arrive at GBDD are available at https://github.com/FAME-GRAPE/GDDB. There are no restrictions on access. The replication codes are accompanied with documentation detailing each step. The codes are distributed as STATA 17 project, do-files and datasets.
